# Olecranon Osteotomy Approach for Complex AO-13C Fractures of Distal Humerus: A Prospective Analysis of 24 Cases

**DOI:** 10.5704/MOJ.1903.005

**Published:** 2019-03

**Authors:** R Singh, H Singh, N Kanodia

**Affiliations:** Department of Orthopaedics, Mayo Institute of Medical Sciences, Uttar Pradesh, India; * Department of Orthopaedics, GSVM Medical College, Kanpur, India; ** Department of Orthopaedics, Sir Ganga Ram Hospital, New Delhi, India

**Keywords:** intra-articular, elbow, lower end humerus, surgical approach, olecranon osteotomy

## Abstract

**Introduction:** Olecranon osteotomy is well described approach for complex intra-articular distal humeral fractures. In this study, we investigated the usefulness and complications of olecranon osteotomy approach for such fractures. We hypothesise that outcome is comparable in young adults and middle age group and also functional outcome is independent of fracture subtype following surgical fixation.

**Materials and Methods:** Between December 2012 and September 2015, twenty-four adult patients (male: 15, female: 9) having mean age of 41.4 years with closed intra-articular fracture (AO-13C) were surgically managed using olecranon osteotomy approach and were followed-up for a mean of 28.5 months (range: 22-35 months). Functional outcome was measured using Mayo Elbow Performance Score (MEPS) and complications were observed. Statistical analysis was done using Student t-test and Kruskal Wallis test.

**Results:** All fractures united by the end of three months. Mean elbow flexion achieved was 123°, mean extension lag was 9° and mean active arc of motion was 114°. Mean MEPS was 87 (excellent: 8, good: 14, fair: 1 and poor: 1). Post-operative transient ulnar nerve palsy was noted in two cases, heterotopic ossification (HO) was in one case, infection in two cases, implant prominence in five and elbow stiffness in three cases. Motion arc was higher in young adults and MEPS was comparable in both age group. Functional outcome was also dependent on fracture subtype. **Conclusion:** The olecranon osteotomy approach for distal humerus fractures had good functional outcome with fewer complications. Joint congruity and fixation could easily be assessed intraoperatively.

## Introduction

Distal humerus fractures although less common, are on an increasing trend over the last few decades^1^. Riseborough and Radin compared conservative and surgical management of these fractures and came to a conclusion in favour of nonsurgical management^2^. However over last few years, with comprehensive understanding of elbow anatomy and newer implant design, enough evidence has been accumulating in favour of surgical fixation. Open reduction and internal fixation (ORIF) of these fractures are now well recognised management. Hence, exposure of fracture fragments to reconstruct anatomy becomes paramount for good outcome. Consequently, the surgical approach becomes very crucial. These fractures have been principally approached from posterior side and various posterior approaches have been mentioned. Various approaches, namely triceps-reflecting anconeus pedicle (TRAP), Bryan and Morrey’s triceps reflecting, and Campbell's triceps-splitting, have been described with pros and cons of each^3-7^. In TRAP and triceps-reflecting approaches, the entire extensor mechanism have to be reflected, have limited exposure and also have well documented triceps weakness and triceps avulsion^3-6^. Triceps splitting allows very limited articular visualisation which makes it undesirable for such injuries^8^. The olecranon osteotomy approach which provides maximum articular surface visualisation, gives better command on fracture fragments and has minimal consequences on extensor mechanism, is often employed for such fracture^9^. However, the olecranon osteotomy approach has additional potential complications such as non-union at osteotomy site, implant issues and resurgeries^8,9^.

In this study, we evaluated the functional outcome of complex intra-articular distal humeral fracture following the olecranon osteotomy approach. We hypothesise that functional outcome is comparable in young adults (≤ 40 years) and the middle-aged group (41-65 years). We also believe that outcome following fracture stabilisation is independent of fracture subtype.

## Materials and Methods

After obtaining ethical committee and departmental review board approval, complex intra-articular fracture of distal humerus in adult patients coming during December 2012 to September 2015 were included in this study. Fractures were classified according to AO classification and only AO 13-C were included. All fractures were operated on by a senior trauma surgeon. Forty-four patients were available for the study and 13 patients were excluded due to three open fractures, eight ipsilateral upper limb fractures, one with associated vascular injury and one pathological fracture. Seven patients lost to follow-up early and who did not return for evaluation, were also excluded. Only patients available for a minimum follow-up of one year were included. Final assessment was done on a total of 24 patients (male: 15, female: 9) with mean age of 41.4 years (range: 20-65 years). Fourteen (53.8%) out of 24 patients had fractures on the right and ten (46.2%) on the left. Fracture occurred in 13 (54.2%) patients in motor vehicle accident, falling on ground in five (28.8%) patients, falling from bicycle in three (12.5%) patients, fall from height in two (8.3%) patients and assault in one (4.2%) patient. As per AO classification, fracture type C1 was in five (20.8%) patients, C2 in seven (29.2%) patients and C3 was in 12 (50%) patients.

**Fig. 1:**
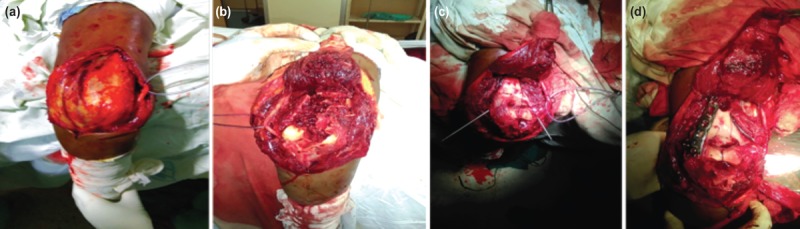
(a) Ulnar nerve was identified and isolated first. (b) Olecranon osteotomy and fracture site exposure. (c) Fracture fragments reduced and temporarily held with K-wires. (d) Fracture internally fixed as per AO principle.

Surgical fixation was done under general anaesthesia in lateral decubitus position with arm support with tourniquet in all patients. Prophylactic antibiotic (cefuroxime 1.5gm) was administered in all cases. Signed informed consent was taken from all patients about fracture type, approach used and possible complications. A uniform surgical technique, a midline posterior incision was used, with slight lateral curve on the olecranon tip to avoid weight-bearing zone. Ulnar nerve was identified, followed by release of the ligament of struthers and medial intermuscular septum to transpose the ulnar nerve anteriorly (Fig. 1a). This was done in all cases in our series. An interval was created between medial intermuscular septum and triceps, and triceps was lifted from the posterior aspect of humerus to create lateral window. The bare area of ulna was identified, which was roughly 2cm. from the olecranon tip, and chevron-shape osteotomy of ulna was done with apex distally. Osteotomy was started with thin oscillating saw and then subchondral bone and articular surface were fractured with a thin osteotome. Dissection was extended proximally as required and the olecranon fragment was wrapped with saline soaked gauge piece, sutured proximally (Fig. 1b,c).

Fracture fragments were exposed completely, small pieces were fitted with each other and temporarily held with K-wires. Headless screws were often used whenever necessary. The definitive fixation of articular surface was done using 4.5mm cannulated screw inserted from lateral to medial direction. Two cannulated screws were preferred to attain rotational stability. This articular fragment was then attached to the condyle and temporarily fixed with K-wires. Fractures sites were stabilised with orthogonal platting: one plate on the medial side and the other on the posterolateral side, roughly perpendicular to each other as per AO principle (Fig. 1d). First, a plate was applied posterolaterally followed by medial platting, roughly perpendicular to each other as per AO principle (Fig. 1d). Fracture fragments were fixed with anatomically contoured locking plates. The olecranon osteotomy was fixed with two 1.8mm (0.072”) smooth K-wires perforating the anterior cortex distal to the coronoid process and stabilised with 18-gauge wire in accordance with tension banding principles. The tips of the K-wires were bent at triceps insertion and impacted to bone. After reduction and fixation, direct visualisation of joint congruity was confirmed, with fluoroscopy to observe joint motion. The wound was closed with suction drain. Post-operatively the elbow was immobilised in 90 degree flexion for two days.

After drain removal at 48 hours, active or assisted range of motion exercises were commenced. The exercises were for 20-30 minutes, three to four times a day initially and gradually increased depending on the patient’s comfort, to achieve up to 90 degree by the end of the second week and full range of motion by two months. Patients were regularly followed-up at six weeks, 12 weeks and thereafter every three months for radiological and functional assessment. Articular step off of more than 2mm or malalignment greater than five degrees in any plane was considered as malunion. Functional assessment was done using Mayo Elbow Performance Score (MEPS). Data were summarised as mean and standard deviation. Categorical data was compared using two-tailed Student t-test. Kruskal Wallis test was used to establish relationship between fracture type and motion arc or extension loss. The p-value <.05 considered for the level of significance for all analysis. The data was analysed using the SPSS version 22 software.

## Results

Mean follow-up of patients was 28.5 months (range: 22-35months). Mean surgical delay was 5.3 days (range: 1-12 days). Fracture and osteotomy site union was radiologically confirmed in all cases. Mean flexion achieved was 123°, extension lag was 9° and active arc of motion was 114°. Range of motion (ROM) was significantly higher in young adults compared to middle age group (t-value: 2.55, p-value: 0.017). No articular step off of more than 2mm or malalingment greater than 5°s was observed in any plane. Range of motion achieved in fracture subtype is summarised (Table I).

**Table I:** Demographic data and functional outcome for olecranon osteotomy

Parameter	Type of fracture			Total
	Type C1	Type C2	Type C3	
Mean age (years)	34.4±5.59	27.42±5.65	52.5±13.60	41.41±13.10
Mean surgical delay (days)	2.6±3.16	3.85±2.94	7.41±3.12	5.37±3.12
Surgical time (minutes)	87±7.58	95±9.57	115..41±9.87	103.45±15.36
Mean flexion (degree)	130±7.07	128.57±6.90	118.33±9.57	123.75±9.69
Mean extension lag (degree)	4±5.47	4.28±4.49	14.58±6.55	9.37±7.70
Mean arc of motion (degree)	126±5.47	124.28±10.17	104.58±10.32	114.79±13.86
Mean mayo elbow performance score (MEPS)	94±8.21	87.85±8.59	83.75±14.00	87.08±11.87

Mean MEPS achieved was 87.08 (excellent: 8, good: 14, fair: 1 and poor: 1) (Table II). On comparison MEPS in these two different age groups was found to be statistically insignificant (t-value: 2.01, p-value: 0.05). Also, the final outcome even after ORIF, was dependent on initial fracture type, Kruskal Wallis test (h-value: 13.35, p-value: 0.001) were significant for fracture type and arc of motion. Extension lag was also statistically significant in fracture subtype (h-value: 11.21, p-value: 0.003).

**Table 2:** Quality analysis of Mayo Elbow Performance Score

Fracture type	MePS			
	Excellent (≥90)	Good (75-89)	Fair (60-74)	Poor (≤59)
Type C1 (n=5)	2 (8.33%)	3 (12.5%)	0	0
Type C2(n=7)	3 (12.5%)	4 (16.67%)	0	0
Type C3(n=12)	3 (12.5%)	7 (29.16%)	1 (4.16%)	1 (4.16%)
Total (n=24)	8 (33.33%)	14 (58.33%)	1 (4.16%)	1 (4.16%)

MEPS=mayo elbow performance score

Major complication in our series was implant prominence in five patients (1: over medial epicondyle, 2: over olecranon and in 2: over both olecranon and medial epicondyle). Transient ulnar nerve palsy occurred in two cases and recovered spontaneously within three months. Heterotrophic ossificans occurred in one patient. Deep seated infection occurred in two patients which subsided with joint debridement and antibiotics (one in each type C2 and C3). Elbow stiffness occurred in three patients and arthrolysis was advised but patients declined in spite of limitation in daily activity.

## Discussion

The optimal surgical approach for distal humerus complex articular fracture is one which provides adequate fracture fragment assessment with minimal tissue disruption. Olecranon osteotomy is conventionally well accepted for distal humeral exposure but has issues related to osteotomy and hardware. In this study, we analysed twenty-four elbows in twenty-four patients with complex intra-articular (AO 13 type C) fractures. We divided the cohort into further subgroups based on fracture configuration (AO classification) and analysed outcome. Mckee and Szako retrospectively analysed 11 elbows with type C fracture managed using olecranon osteotomy approach and reported net arc of motion of 102.7°^10^. Also, Ljungquist *et al* in their systematic review detected mean arc of motion after olecranon osteotomy to be 107°^11^. In our series, we were able to achieve 114° of mean active arc of motion which is close to those reported findings,

Complications with olecranon osteotomy include implant prominence or failure in 27-80% and non-union in 0-15% cases^12-15^. Non-union of osteotomy site was reported as high as 30% when transverse osteotomy was done^16^. We performed chevron-type osteotomy in all our patients on the basis of its larger contact area and better rotational stability compared to transverse osteotomy^17^. In comparing fixation method for chevron olecranon osteotomy, Wagener *et al* found bicortical purchase was achieved with two K-wires with tension band wiring and with intramedullary cancellous screw with tension band, both providing enough elbow stability for daily use^17^. We utilised two bicortical K-wires and tension band construct to fix our osteotomy site in all cases. We did not experience any non-union issues in our series. Another technique to fix olecranon osteotomy is with plates and screws. However, plating in olecranon fracture became less popular because of necessity for wide surgical exposure, bulky and expensive implants and also implant removal often becomes unavoidable^18^. Furthermore, using a plate to fix osteotomy site poses significant wound complications compared to tension band^19^. Also, Coles *et al* in their six-year experience with chevron olecranon osteotomy did not report any non-union. The key factor they concluded was to secure stabilisation of osteotomy site rather than fixation type^9^. Implant prominence was the most common complication in our series and was noted in 20.8%. Prominence was typically noted in the epicondylar area of the humerus and also in olecranon tip. But in spite of risk of non-union implant complication and wound dehiscence, Wilkinson and Stanley advocate olecranon osteotomy while dealing with intra-articular fracture^8^.

We routinely transpose ulnar nerve anteriorly to reduces the impingement between nerve and implants during elbow motion. Though there were no consistent reports supporting the routine transposition of ulnar nerve, we did it to avoid any influence on the result. Currently, a study on this subject entitled "A Multicentre, Randomized Trial of Simple Decompression *versus* Anterior Transposition of the Ulnar Nerve for Acute, Displaced Fractures of the Distal Humerus Treated With Plate Fixation” is ongoing and the results are awaited^20^. We encountered two patients (8.3%) with transient ulnar nerve injury. The probable cause is traction injury to the nerve during surgery. The incidence of heterotropic ossification (HO) which is a well-established sequelae of elbow trauma, has been reported incidence as high as 89% especially with periarticular elbow fracture with associated traumatic head injury^21,22^. The role of the surgical approach in the development of HO is still controversial. Chen *et al* reported 12% cases of HO when distal humerus fracture was treated with olecranon osteotomy compared to negligible HO in triceps-sparing approach^23^. Abrams *et al* stated no noteworthy difference in HO in olecranon osteotomy or triceps-sparing approach^24^. Hong *et al* noted that duration of surgery, timing of surgery and fracture dislocation were independent risk factors for development of HO but did not comment upon the role of surgical approach in developing HO^25^. In a systematic review, Ljungquist *et al* observed that HO occurred in four out of total 66 patients of such fractures treated with olecranon osteotomy and none in triceps sparing group^11^. We observed 4.2% incidence of HO in our series. We did not use any prophylaxis for HO fearing risk of non-union. With lack of clear evidence, it is difficult to conclude the role of surgical approach in HO development.

Surgical delay is also considered an important parameter in outcome. Delay in surgical intervention leads to soft tissue contracture and limits functional motion arc^26^. But there are conflicting reports about surgical delay versus motion arc and MEPS. Erpelding *et al* reported no significant co-relation between surgical delay and motion arc or MEPS^27^. Further, Elmadag *et al* claimed to obtain good to excellent functional outcome if patients were operated on in less than three days after trauma, although the authors did not comment further on statistical co-relation between surgical delay and outcome^28^. We did notice a weak negative correlation between surgical delay and age against performance score and functional outcome, but it was not statistically significant. Also, Chen *et al* reported no statically significant correlation between age against MEPS and motion arc, but did not mention the p-value^23^.

In view of the limitations of the study, first, there was absence of control groups and it was a small study with limited number of patients. Secondly, longer follow up is required to look for long-term result of osteotomy surgical approach. Thirdly, we did not include geriatric population, so drawing an authoritative conclusion on age versus outcome is not viable. Lastly, though we did not find any subjective difference in elbow extension compared to the contralateral elbow, we did not objectively investigate extension strength. Though the main objective of our study was to assess functional outcome, we still consider failure to investigate this as a limitation.

## Conclusion

Olecranon osteotomy for intra-articular fracture of distal humerus has high rate of healing and good functional outcome with fewer complications. Joint congruity can be assuredly restored and fixation can be comfortably assessed intraoperatively.
